# Prevalence and Predictors of Malnutrition among Guatemalan Children at 2 Years of Age

**DOI:** 10.1371/journal.pone.0164772

**Published:** 2016-11-02

**Authors:** Jason M. Nagata, James Gippetti, Stefan Wager, Alejandro Chavez, Paul H. Wise

**Affiliations:** 1 Department of Pediatrics, Stanford University, Palo Alto, California, United States of America; 2 Center for Health Policy and the Center for Primary Care and Outcomes Research, Stanford University, Palo Alto, California, United States of America; 3 Graduate School of Business, Stanford University, Palo Alto, California, United States of America; Hospital Universitario de la Princesa, SPAIN

## Abstract

**Objective:**

To identify the prevalence and predictors of malnutrition among 2-year old children in the Western Highlands of Guatemala.

**Methods:**

Prospective cohort of 852 Guatemalan children in San Lucas Toliman, Guatemala followed from birth to age 2 from May 2008 to December 2013. Socio-demographic, anthropometric, and health data of children was collected at 2 month intervals.

**Results:**

Among the 402 males and 450 females in the cohort, mean weight-for-age Z-score (WAZ) declined from -0.67 ± 1.01 at 1 year to -1.07 ± 0.87 at 2 years, while mean height-for-age Z-score (HAZ) declined from -1.88 ± 1.19 at 1 year to -2.37 ± 0.99 at 2 years. Using multiple linear regression modeling, number of children <5 years old, vomiting in the past week, fever in the past week, and WAZ at 1 year were significant predictors of WAZ at 2 years. Significant predictors of HAZ at 2 years included household size, number of children <5 years old, diarrhea in the past week, WAZ at 1 year, and HAZ at 1 year. Vomiting in the past week and WAZ at 1 year were significant predictors of weight-for-height z-score (WHZ) at 2 years.

**Conclusions:**

Number of children <5 years old, symptoms such as vomiting or diarrhea in the previous week, and prior nutritional status were the most significant predictors of malnutrition in this cohort. Future research may focus on the application of models to develop predictive algorithms for mobile device technology, as well as the identification of other predictors of malnutrition that are not well characterized such as the interaction of environmental exposures with protein consumption and epigenetics.

## Introduction

Child malnutrition remains a major public health challenge in low- and middle-income countries. Globally, 73 million children live with moderate or severe acute malnutrition [1]. More than half of the 11 million deaths of children under five are attributable to malnutrition, which may cause increased susceptibility to infectious disease [1,2]. Malnourished children experience increases in chronic illnesses and disabilities, have impaired brain function, and suffer from loss of physical and economic productivity [1,3].

In low- and middle-income countries including Guatemala, growth failure has been observed in the first 24 months, which is more pronounced for length than for relative weight [4,5]. Height for age z-score (HAZ) has been shown to start with a deficit at birth and declines further until the age of 2 until reaching an apparent nadir [6,7]. Therefore, the time period -9 to 24 months has been identified as a “critical window” of opportunity to focus growth promoting interventions [6]. This window has served as a rallying point for global initiatives, and been marketed as the “first 1000 days.” Global progress has been made as the proportion of stunted children has decreased from 40% to 25% from 1990 to 2012, although improvements have been unequally distributed throughout different regions [8]. In addition, determinants of malnutrition can vary greatly by region; therefore, interventions may be best tailored to local epidemiologic factors [9,10].

Previous studies have examined predictors of child malnutrition in low- and middle-income countries. Poverty and morbidity are strongly associated with child malnutrition, through complex relationships [1,11]. Other predictors of child malnutrition identified in previous literature include parental education levels, with maternal education more strongly predictive than paternal education, social deprivation, number of children in household, and sources of drinking water [1]. Previous literature in the Western highlands of Guatemala found that significant predictors of low HAZ at 3 months were low newborn HAZ, male sex, and the interaction between maternal education and maternal age [3].

Identifying predictors of malnutrition is crucial for the allocation of nutritional interventions during the “critical window,” particularly given limited resources. The Instituto de Nutricion de Centroamerica y Panama (INCAP) nutrition interventions in four Guatemalan villages demonstrated that nutritional supplementation in children improved their growth and development through adolescence and additionally their future offsprings’ nutritional status [12,13]. A systematic review of 29 efficacy and 13 effectiveness trials of complementary feeding interventions during early childhood in developing countries have shown a mean effect size of 0.26 (range -0.02 to 0.57) for weight for age z-score (WAZ) [14]. In addition, mobile technology may be a novel platform to improve screening accuracy for child malnutrition in community-based models [15].

**Fig 1:**
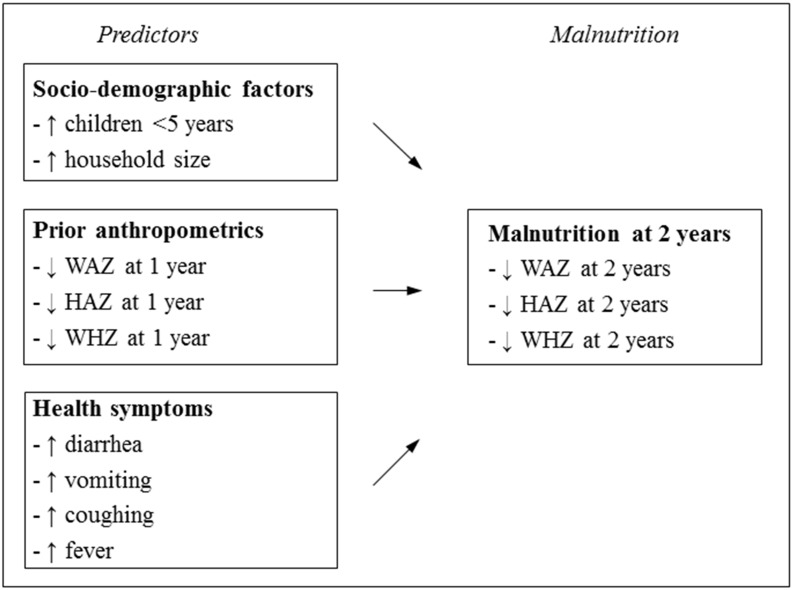
Conceptual framework for predictors of malnutrition among Guatemalan children at 2 years of age

In this study, we identify the prevalence and predictors of child malnutrition in Guatemala for potential application to develop predictive algorithms for mobile devices to help identify children at greatest risk for falling into serious malnutrition. We also examine the extent to which anthropometrics at previous visits predict malnutrition. We hypothesize that malnutrition at 1 year of life, infectious symptoms such as fever or diarrhea, and larger household size, will predict malnutrition at 2 years of age. A conceptual framework for the analysis is illustrated in Fig 1.

## Materials and Methods

### Study Setting

Guatemala has the highest rate of child stunting in the Americas [16], estimated at 47%, 53%, and 56% at birth, three, and six months of age, respectively [3]. In addition to high rates of stunting and undernutrition, Guatemala is facing an increasing prevalence of obesity/overweight and metabolic syndrome, leading to a double burden of malnutrition [17–19]. Access to clean water remains a considerable health challenge in the region and contributes to a significant burden of water-borne diseases such as diarrhea, dysentery, and intestinal parasites particularly among children [20].

### Sample and Data collection methods

Children from 20 communities surrounding San Lucas Tolimán in the Western Highlands of Guatemala were recruited for the study program by community health promoters from May 2008 to December 2013. Community health promoters live in the communities they serve and stay informed about pregnant mothers and the birth of new children. Shortly after birth, community health promoters visit the homes of the mother to invite them to join the program. In addition, community health promoters work closely with the local health center (*Centro de Salud*) and identify newborns at the health centers when they have the newborn health visit or for immunizations. The refusal rate among approached mothers was less than 1%. Community health promoters underwent a rigorous training program with certification that included training on taking standardized and accurate height and weight measurements.

Socio-demographic information of children and their parents was collected upon enrollment including household size, paternal occupation, and maternal marital status. Children from birth to age 24 months had a health assessment every 2 months which included standardized measurements of their weight and height. Prior to measurement of anthropometry, children’s shoes were removed so they were only wearing a diaper or light clothes, the weighing scale was calibrated, and two promoters were involved in the measurement of height to ensure the child was in the recumbent position with their body as straight as possible. Weight and height measurements were repeated if a large change was noted from the visit two months prior. Information on parental report of child fevers, diarrhea, vomiting, or cough symptoms in the previous week was also collected. Anthropometric measurements were converted into weight-for-age z-score (WAZ), height-for-age z-score (HAZ), and weight-for-height z-score (WHZ) according to WHO child growth standards [21].

Incaparina, a nutrition supplement made from maize and soy flour, was provided to children over 6 months with WAZ < -2.5 based on measurements from the community health promoters. If a child met the supplementation threshold (WAZ <-2.5) in the Centro de Salud, a community health promoter conducted a home visit to confirm the measurement and discuss the commitment of receiving the supplement, which included weekly home visits. If a child met the supplementation threshold during a home visit, the health promoter initiated the discussion of the supplementation program at the home visit. Community health promoters provided the child with a one month supply of the Incaparina, provided instruction to the family on how to prepare the Incaparina, observed the family prepare the Incaparina, and directly observed the consumption of the Incaparina by the child during the home visit. However, direct observation of preparation and consumption only occurred during the weekly home visit, and not at all meals. Children between 6 and 12 months received approximately 50 kcal/day of Incaparina and children older than 12 months received approximately 100 kcal/day of Incaparina. Monthly provisions of Incaparina were given at the home visit and then subsequently at the beginning of each month. Findings from an evaluation of the effect of the nutrition supplementation program in this community are reported elsewhere [22].

### Ethical Considerations

This study was approved by the Stanford Institutional Review Board and the Review Committee of the Hospital Obras Sociales do Monsignor Gregorio Schaffer, San Lucas Toliman. Parents/guardians of children received an explanation of the program and provided their written informed consent for their child’s participation in the program via a signed form that was approved by the institutional review board. Patient information was de-identified and anonymized prior to data analysis.

### Data Analysis

Quantitative statistical analyses were conducted with R (R Foundation for Statistical Computing, Vienna, Austria). Simple and multiple linear regression were performed using WAZ, HAZ, and WHZ at 2 years as the continuous dependent variables and sex, household size, number of children <5 years old, diarrhea in past week, vomiting in past week, fever in past week, coughing in past week, WAZ at 1 year, HAZ at 1 year, and WHZ at 1 year as independent variables. The Benjamini-Hochberg procedure was used to adjust for a false discovery rate given multiple statistical tests, using an alpha of 0.1 [23]. Frequency plots were constructed using WAZ, HAZ, and WHZ scores at 1 and 2 years of age. The Z-scores were rounded to the nearest 0.5. The size of the plot represented the size of the transition cohort and the bubble color signified a positive (green), negative (red), or neutral (grey) change from year 1 to 2.

## Results

Overall, 852 children were included in the study, of which 52.8% were female (Table 1, S1 Table). Average household size was 6.12 ± 2.79 with an average of 1.70 ± 0.69 children under 5 years old. Nearly forty percent of children had mothers who were married. Mean WAZ declined from -0.67 ± 1.01 at 1 year to -1.07 ± 0.87 at 2 years, while mean HAZ declined from -1.88 ± 1.19 at 1 year to -2.37 ± 0.99 at 2 years. Children had symptoms of diarrhea (0.82 ± 1.86 days), vomiting (0.12 ± 0.65 days), coughing (0.99 ± 2.03 days), and fever (0.54 ± 1.43 days) in the past week.

**Table 1:** Selected socio-demographic, health and socio-behavioral characteristics of study participants.

	n	Mean	SD	Range
*Demographics*				
Sex (%)				
Male	402	47.2%		
Female	450	52.8%		
Mother's marital status (%)				
Married	339	39.9%		
Living with partner	462	54.4%		
Single	41	4.8%		
Widowed	7	0.8%		
Age of mother (years)	767	26.7	6.8	14–48
Age of father (years)	723	29.76	7.36	17–61
Number of children <5 years old	849	1.7	0.69	0–4
Total number in household	850	6.12	2.79	1–17
*Anthropometrics*				
WAZ at 1 year	851	-0.67	1.01	
HAZ at 1 year	851	-1.88	1.19	
WHZ at 1 year	852	0.46	1.08	
WAZ at 2 years	852	-1.07	0.87	
HAZ at 2 years	852	-2.37	0.99	
WHZ at 2 years	852	0.25	0.88	
*Symptoms*				
Days of diarrhea in past week	849	0.82	1.86	0–7
Days of vomiting in past week	850	0.12	0.65	0–7
Days of coughing in past week	850	0.99	2.03	0–7
Days of fever in past week	849	0.54	1.43	0–7

WAZ = weight-for-age z-score; HAZ = height-for-age z-score; WHZ = weight-for-height z-score

Using simple linear regression, significant individual predictors of WAZ, HAZ, and WHZ at 2 years included female sex, household size, number of children <5 years old, diarrhea in the past week, WAZ at 1 year, and HAZ at 1 year (Tables 2, 3 and 4). Number of children <5 years old, vomiting in the past week, fever in the past week, and WAZ at 1 year were significant predictors of WAZ at 2 years in the multiple linear regression model (Table 2). Using the Benjamini-Hochberg procedure, number of children <5 years old (t -2.62, p = 0.01) and vomiting in the past week (t = 2.69, p = 0.01) emerged as the most significant predictors of WAZ at 2 years. Significant predictors of HAZ at 2 years included household size, number of children <5 years old, diarrhea in the past week, WAZ at 1 year, and HAZ at 1 year in the multiple linear regression model (Table 3). Using the Benjamini-Hochberg procedure, household size (t = -2.14, p = 0.03), number of children <5 years old (t = -2.38, p = 0.02) and diarrhea in the past week (t = -3.05, p<0.01) were the most significant predictors of HAZ at 2 years. Significant predictors of WHZ at 2 years included vomiting in the past week and WAZ at 1 year in the multiple linear regression model (Table 4). Using the Benjamini-Hochberg procedure, vomiting (t = 2.21, p = 0.03) was the most significant predictor of WHZ at 2 years.

**Table 2:** Results from simple and multiple linear regressions with WAZ at 2 years as the dependent variable and various demographic, anthropometric, and clinical characteristics as independent variables.

	Model 1	Model 2	Model 3	Model 4	Model 5	Model 6	Model 7	Model 7	Model 7	Model 8
Female sex	-0.19 (0.06)**									0.01 (0.04)
Children <5 in household		-0.16 (0.04)***								-0.09 (0.03)**
Total number in household			-0.04 (0.01)**							-0.002 (0.01)
WAZ at 1 year				0.64 (0.02)***						0.62 (0.03)***
HAZ at 1 year					0.35 (0.02)***					0.04 (0.02)
Days of diarrhea in past week						-0.04 (0.02)*				-0.02 (0.01)
Days of vomiting in past week							-0.04 (0.05)			0.09 (0.03)**
Days of coughing in past week								-0.01 (0.02)		-0.01 (0.01)
Days of fever in past week									-0.03 (0.02)	0.03 (0.02)*
Observations	852	849	850	851	851	849	850	850	849	842
R^2^	0.01	0.01	0.01	0.56	0.24	0.01	0.00	0.00	0.00	0.57
Adjusted R^2^	0.01	0.01	0.01	0.56	0.23	0.01	0.00	0.00	0.00	.57

B (SE) presented for each model

*p<0.05;

**p<0.01;

***p<0.001

**Table 3:** Results from simple and multiple linear regressions with HAZ at 2 years as the dependent variable and various demographic, anthropometric, and clinical characteristics as independent variables.

	Model 1	Model 2	Model 3	Model 4	Model 5	Model 6	Model 7	Model 7	Model 7	Model 8
Female sex	-0.22 (0.07)**									-0.01 (0.05)
Children <5 in household		-0.25 (0.05)***								-0.10 (0.04)*
Total number in household			-0.07 (0.01)***							-0.03 (0.01)*
WAZ at 1 year				0.57 (0.03)***						0.32 (0.03)***
HAZ at 1 year					0.52 (0.02)***					0.35 (0.03)***
Days of diarrhea in past week						-0.06 (0.02)***				-0.05 (0.02)**
Days of vomiting in past week							-0.10 (0.05)			0.04 (0.01)
Days of coughing in past week								0.01 (0.02)		0.01 (0.01)
Days of fever in past week									-0.02 (0.02)	0.02 (0.02)
Observations	852	849	850	851	851	849	850	850	849	842
R^2^	0.01	0.03	0.04	0.31	0.40	0.01	0.00	0.00	0.00	.49
Adjusted R^2^	0.01	0.03	0.03	0.33	0.40	0.01	0.00	0.00	0.00	.48

B (SE) presented for each model

*p<0.05;

**p<0.01;

***p<0.001

**Table 4:** Results from simple and multiple linear regressions with WHZ at 2 years as the dependent variable and various demographic, anthropometric, and clinical characteristics as independent variables.

	Model 1	Model 2	Model 3	Model 4	Model 5	Model 6	Model 7	Model 7	Model 7	Model 8	Model 9
Female sex	0.15 (0.06)*										0.07 (0.05)
Children <5 in household		-0.04 (0.04)									0.04 (0.04)
Total number in household			0.001 (0.01)								0.02 (0.01)
WAZ at 1 year				0.46 (0.03)***							0.70 (0.25)**
HAZ at 1 year					0.10 (0.03)***						-0.26 (0.14)
WHZ at 1 year						0.43 (0.02)***					-0.07 (0.19)
Days of diarrhea in past week							-0.01 (0.02)				0.01 (0.02)
Days of vomiting in past week								0.02 (0.05)			0.09 (0.04)*
Days of coughing in past week									-0.03 (0.02)		-0.02 (0.01)
Days of fever in past week										-0.03 (0.02)	0.02 (0.02)
Observations	852	849	850	851	851	852	850	850	850	849	842
R^2^	0.00	0.00	0.00	0.28	0.02	0.28	0.00	0.00	0.00	0.00	.49
Adjusted R^2^	0.00	0.00	0.00	0.28	0.02	0.28	0.00	0.00	0.00	0.00	.48

B (SE) presented for each model

*p<0.05;

**p<0.01;

***p<0.001

**Fig 2:**
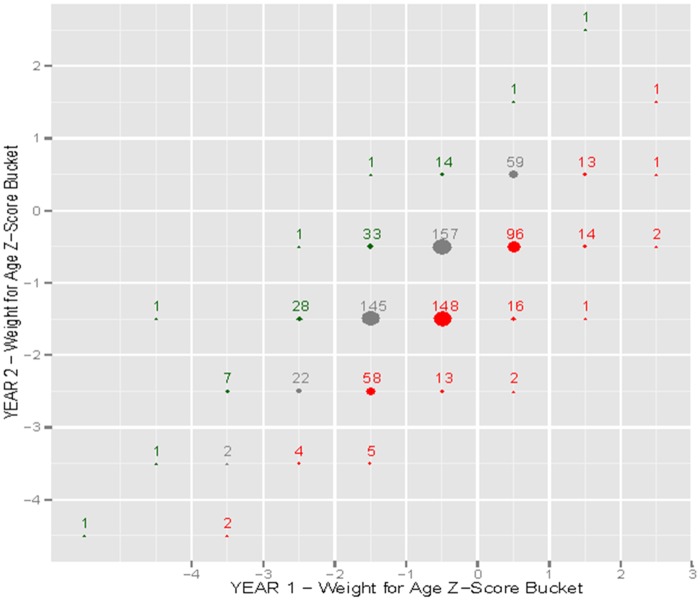
Frequency plot of weight-for-age z-score (WAZ) from 1 to 2 years of age. The size of the plot represents the size of the transition cohort and the bubble color signifies a positive (green), negative (red) or neutral (grey) change in z-score from year 1 to 2.

**Fig 3:**
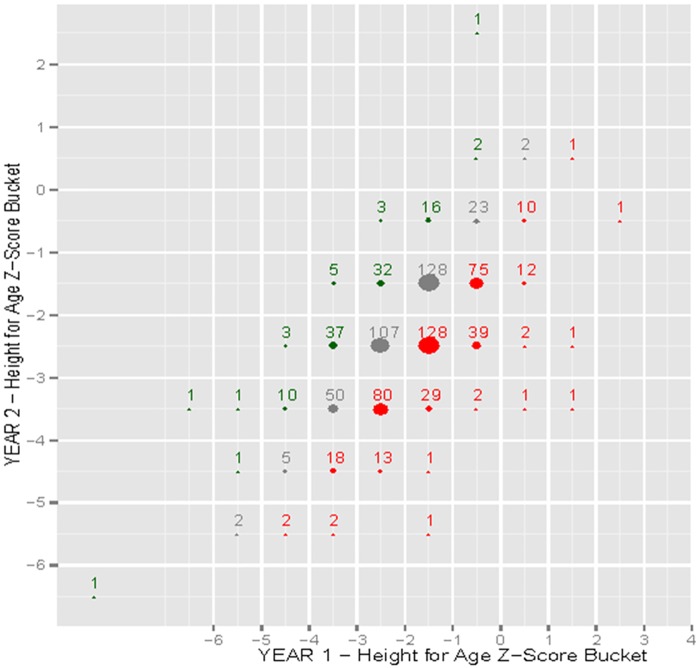
Frequency plot of height-for-age z-score (HAZ) from 1 to 2 years of age. The size of the plot represents the size of the transition cohort and the bubble color signifies a positive (green), negative (red) or neutral (grey) change in z-score from year 1 to 2.

**Fig 4:**
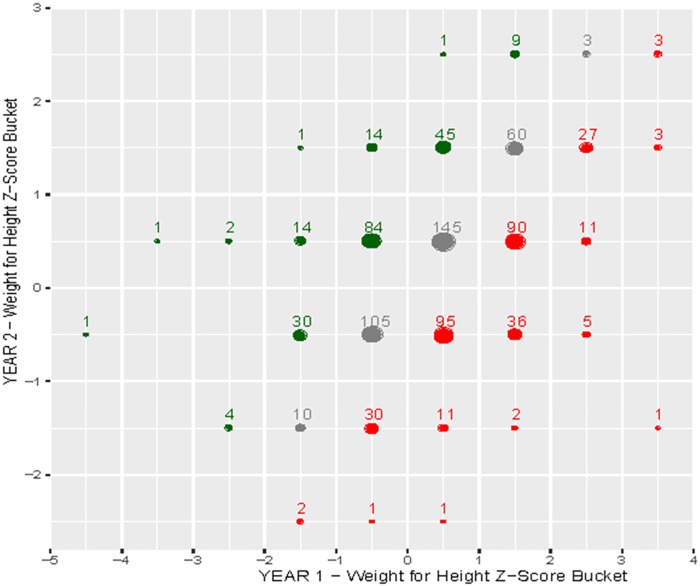
Frequency plot of weight-for-height z-score (WHZ) from 1 to 2 years of age. The size of the plot represents the size of the transition cohort and the bubble color signifies a positive (green), negative (red) or neutral (grey) change in z-score from year 1 to 2.

Frequency plots show WAZ (Fig 2), HAZ (Fig 3), and WHZ (Fig 4) trends from 1 to 2 years of age. The size of the plot represents the size of the transition cohort and the bubble color signified a greater frequency of decreased Z-scores (red) rather that increased Z-scores (green) from 1 to 2 years of age.

## Discussion

We report a larger degree of stunting and less severe degree of underweight in our cohort of Guatemalan children. Mean HAZ, WAZ, and WHZ worsened from 1 to 2 years despite nutritional interventions for children with WAZ < -2.5. Significant predictors of underweight included previous WAZ, number of children <5 in household, and symptoms of vomiting or fever in the past week. Significant predictors of stunting included previous HAZ and WAZ, household size, number of children <5 in the household, and diarrhea in the past week. Significant predictors of wasting included vomiting in the past week and previous WAZ.

Guatemalan children had more pronounced stunting than underweight through age 2. Furthermore, both HAZ (-1.88 to -2.37) and WAZ (-0.67 to -1.07) worsened from 1 to 2 years. This is consistent with trends previously reported in Guatemala and in other regions [6,7]. One previous Guatemalan study demonstrated that stunting reached a nadir at 24 months of age before starting to regain HAZ from 24 to 48 months [7]. Despite nutritional interventions for children with WAZ < -2.5 in the first 2 years of life, nutritional status nonetheless worsens during this time period. This reinforces the idea of the critical window from -9 to 24 months for nutritional interventions. A previous study in this population found a strong age dependence in the impact of supplementary food on WAZ and HAZ, with the benefits decreasing from 6 months to 20 months of age [22]. A simulation study based on a statistical model estimated that focusing allocation of nutrition supplementation to infants could reduce underweight severity by 13.6–14.1% and the stunting severity by 7.1–8.0% [22].

One of the most significant predictors of malnutrition at 2 years was prior malnutrition. Previous WAZ was the most significant predictor of WAZ at 2 years, previous HAZ and WAZ were significant predictors of HAZ at 2 years, and previous WAZ was the most significant predictor of WHZ at 2 years. This is consistent with one previous study in the Western Highlands of Guatemala which demonstrated that newborn HAZ was a significant predictor of HAZ at three months [3] and similar findings have been documented in Malawi where small size in the first three months of life predicted low WAZ later in childhood [10].

Household size, particularly number of children <5 in the household, was a significant predictor of both WAZ and HAZ. This is consistent with previous literature demonstrating that number of children in household predicted WAZ and other indicators of malnutrition in Bangladesh [1], Vietnam [24], and Somalia [25]. Families with more children may experience more economic strain due to increased food expenditure and may have less time to devote to each child [24]. Therefore, increasing birth intervals between offspring and ensuring access to family planning for mothers are strategies that has been suggested to improve nutritional status of children [1,26].

We found that infectious symptoms, such as diarrhea, vomiting, and fever are significant predictors of malnutrition. Strong links between infection and malnutrition are well established [27], particularly the bidirectional relationship between malnutrition and diarrhea. Diarrhea can lead to malnutrition through malabsorption, reduced intake of nutrients, fecal nutrient losses, and an inflammatory response [28]. On the other hand, malnutrition can lead to a less robust immune response making children more susceptible to enteric and other infections [29]. Children with diarrhea symptoms in the past 2 weeks had an increased risk of wasting (35% increase) and stunting (29% increase) in Somalia [25] and similar results have been shown in Malawi [10]. In the medical evaluation of children with symptoms such as diarrhea, vomiting, and fever concerning for infection, health workers may concurrently evaluate for malnutrition and the need for nutritional supplementation particularly during times of acute illness [30]. Community-based malnutrition programs have started to explore the use of novel mobile technology devices to improve the accuracy of identifying children at risk for malnutrition [15].

This study has several limitations. The non-randomized nature of this observational study precludes the ability to prove causality. If the purpose of the analysis were to establish causal relationships or to fit coefficients into a structural equations model, there may be a problem with endogeneity as there may be unobserved conditions/characteristics that made children candidates for supplementation that were also highly correlated with HAZ, WAZ, and WHZ; however, this was not the goal of the analysis. Measurement error and lack of standardization may lead to limitations in data quality and may underestimate predictors as anthropometric measurements were not routinely taken in duplicate or triplicate; however, measurements with large changes from previous measurements were rechecked. Parents were provided instruction on the preparation of the nutrition supplement and direct observation of the parents’ preparation and child’s consumption occurred during the weekly home visit with the health promoter; however, not all meals were monitored to ensure proper preparation and exclusive consumption by the child it was intended for. Prior studies have shown that nutritional supplements for malnutrition may be shared across family members even if only intended for one individual [31]; therefore, programs may consider providing supplements for all children in households with an undernourished child or experiencing food insecurity. We did not collect data on whether or not families were concurrently receiving supplementation from additional sources. We do not analyze in detail the effects of the supplementation program as these results are reported elsewhere [22]. The data were collected in a community in the Western Highlands of Guatemala with a large indigenous Maya population and may not be generalizable to other regions of the world. Furthermore, health promoters had discretion to feed children deemed to be sick upon their home visit, so some children who did not meet strict WAZ <-2.5 may have received nutrition supplementation. However, we find that illness symptoms (diarrhea, vomiting, fever) were important predictors of malnutrition. If promoters were not given discretion to feed sick children at home visits, there may have been incentive to alter their WAZ data to allow them to qualify for supplementation. Furthermore, we did not have a control group for nutrition supplementation. Strengths of this study include its large sample size and longitudinal data collected at regular intervals throughout participants’ first 2 years of life.

## Conclusions

The present study identifies worsening deficits in stunting and underweight in a cohort of Guatemalan children over their first 2 years of life, despite nutritional intervention. Significant predictors of underweight included previous WAZ, number of children <5 in household, and symptoms of vomiting or fever in the past week. Significant predictors of stunting included previous HAZ and WAZ, household size, number of children <5 in the household, and diarrhea in the past week. Significant predictors of wasting included vomiting in the past week and previous WAZ. Future research could use regression models to develop predictive algorithms for application to mobile devices to help identify children at greatest risk for falling into serious malnutrition. In addition, future studies could identify other predictors of malnutrition that are not well characterized such as the interaction of environmental exposures with protein consumption and epigenetics.

## Supporting Information

S1 Table Supporting data used in analysis. (CSV)
